# Clinicians and AI use: where is the professional guidance?

**DOI:** 10.1136/jme-2022-108831

**Published:** 2023-08-22

**Authors:** Helen Smith, John Downer, Jonathan Ives

**Affiliations:** 1 Centre for Ethics in Medicine, Population Health Sciences, University of Bristol, Bristol, UK; 2 School of Sociology, Politics and International Studies, University of Bristol, Bristol, UK

**Keywords:** information technology, Medical Ethics

## Abstract

With the introduction of artificial intelligence (AI) to healthcare, there is also a need for professional guidance to support its use. New (2022) reports from National Health Service AI Lab & Health Education England focus on healthcare workers’ understanding and confidence in AI clinical decision support systems (AI-CDDSs), and are concerned with developing trust in, and the trustworthiness of these systems. While they offer guidance to aid developers and purchasers of such systems, they offer little specific guidance for the clinical users who will be required to use them in patient care.

This paper argues that clinical, professional and reputational safety will be risked if this deficit of professional guidance for clinical users of AI-CDDSs is not redressed. We argue it is not enough to develop training for clinical users without first establishing professional guidance regarding the rights and expectations of clinical users.

We conclude with a call to action for clinical regulators: to unite to draft guidance for users of AI-CDDS that helps manage clinical, professional and reputational risks. We further suggest that this exercise offers an opportunity to address fundamental issues in the use of AI-CDDSs; regarding, for example, the fair burden of responsibility for outcomes.

## Introduction

In the UK, national regulators are planning for artificial intelligence (AI) use by developing context-specific regulation underpinned by a core set of principles.^1^ This generalist approach is far from ready for implementation, however, and will likely be too broad to apply to specialist use-cases. In the interim, therefore, preparations are being made for the specific introduction of AI in healthcare delivery. This has the potential to have a high impact on health services. In this context, AI is often considered in terms of supporting clinical decision-making: operationalised as ‘clinical decision support systems’ (CDSSs).^2^

While CDSSs are not in themselves new, new challenges arise as they come to incorporate increasingly complex AI processes. Where adopted, their use will be a novel addition to the traditional model of multidisciplinary team working, and amount to a monumental change in practice for clinicians. Such a quantum leap in clinical practice will need to be supported by leading healthcare bodies to ensure patient safety.

The goal of bringing AI to use in the UK National Health Service (NHS) has been pursued rapidly by organisations such as the Department of Health and Social Care,^3^ NHSX^4^ and the National Institute of Clinical Excellence (NICE).^5^ To date, such work has led to the development of guidance for those who develop or purchase AI for clinical use,^6–8^ but has largely neglected the needs of those who will be using it for patient care.

This trend can be seen in two (2022) reports, jointly authored by NHS AI Lab & Health Education England (HEE).^9 10^ These reports (henceforth referred to here as ‘report 1’ and ‘report 2’) are concerned with the development of trust, trustworthiness and ultimately the confidence clinical users (can) have in AI. Trust and confidence in the technology must necessarily be fostered if AI adoption is to be successful, but this does not constitute the whole picture. Not least because, in addition to being cognisant of issues around patient safety, clinical users will need to look beyond the physical risks that AI might pose to patients and attend to the professional and organisational risks it might pose to the users themselves. A clinical user’s relationship towards AI-CDSS use as a clinical tool should be shaped by guidance from users’ regulatory and professional bodies (eg, Royal Colleges and unions of the clinical professions). At the time of writing, however, little professional guidance has been offered by these bodies and certainly none that is comprehensive, united and cross-professional.

This paper is a response to HEE’s reports 1 and 2. It outlines the need for—and importance of—a consensus on professional, ethical and legally justifiable principles into one overarching item of guidance that specifically addresses the use of AI-CDSS in all clinical practice. It further speculates on the optimal contents of that guidance.

### The need for, and importance of, professional guidance

Healthcare provision has historically been the domain of clinicians, with plans of care being decided between them and their patients (within constraints set by regulators). Clinicians have always had the option of referring to textbooks or conferring with colleagues to inform their decision, but the task of processing the information from these sources, and proposing a treatment strategy, fell to the clinician alone. The adoption of AI-CDSSs in clinical decision-making has the potential to bring about a step-change here, insofar as the AI-CDSS could take over that processing and strategy developing role, with the clinician being the gatekeeper who says ‘yes’ or ‘no’ to the AI-CDSS’s proposal. While this means that, certainly for the foreseeable future, AI-CDSSs will require oversight from experienced, competent and knowledgeable clinicians, their presence has the clear potential to disrupt the clinical space. This disruption could be particularly trenchant if the AI-CDSS has ‘evolving functionality’, which means it can adapt both what it does, and how it does it, in response to external stimuli. This makes the system harder to predict, and next to impossible to understand, meaning that the clinical gatekeeper has to oversee a system with entirely opaque decision-making processes. As such, trust, trustworthiness and confidence in the AI-CDSS is essential for its use, but likely hard to come by. Thus, guidance about what can be trusted, in what circumstances and to what extent is essential.

Clinical users are acknowledged in report 2 as one of five ‘archetypes’ to be considered in developing healthcare workers’ confidence in AI. The other archetypes identified are shapers, drivers, creators and embedders.^1^ The governance needs of these other four archetypes are being met in the form of guidance (and other sources of advice) for those who wish to develop, sell and purchase AI destined for clinical use.^3–8^ These resources signpost regulatory needs and guide the ethical creation, selection and deployment of AI-CDSS for healthcare, but they neglect the needs of the ultimate end users—clinical users of AI-CDSSs.

Clinicians’ conduct has long been subject to professional regulation, with clinical roles supported by robust professional guidance that can be either something generic to the profession or focused on very specific areas of practice. General professional standards of conduct are set by the clinical professional regulators, which stipulate the expected minimum behaviours expected of clinical professionals—towards patients, the public and each other—and are delivered as a code of professional conduct, for example, General Medical Council,^11^ Nursing and Midwifery Council,^12^ Health and Care Professions Council.^13^ Where a clinician is found to have breached professional standards, they may be held to account by their regulatory body and face consequences such as restriction or suspension of practice, or at the extreme, their removal from their professional register, which will prevent them from practising again.^14^ Professional guidance can come from bodies other than regulators, such as the Royal Colleges, and while such guidance does not have the legal bite of regulation it often occupies ‘quasi-legislative’ status, insofar as it defines ‘good practice’ standards against which the profession is judged.^15^ This is exemplified in the plethora of professional ethical guidance published by multiple clinical bodies during first wave of COVID-19.^16^ The interplay of professional regulation and professional guidance is well established and ensures a predictable and (for the most part) consistent standard of care and professionalism in healthcare services. This is important as it promotes public confidence in healthcare, thus permitting vulnerable patients to place trust in clinicians.

Any future introduction and adoption of AI-CDSSs to clinical decision-making will require that these structures be re-examined. This is because its presence would introduce a new (non-human) actor into the theatre of healthcare, which is not currently accommodated by professional regulation and for which professional guidance is lacking. The introduction of AI-CDSSs into healthcare decision-making disrupts the usual practice of deliberation between clinician, patient and (where relevant) clinical colleagues in a way that problematises current understandings of responsibility and accountability; an AI-CDSS is not a professionally regulated individual who can be held accountable or responsible for its actions.

Consequently, AI-CDSS use in healthcare decision-making complicates the lines of influence in a patient’s care planning and makes already complex questions regarding professional accountability and responsibility (for the outcomes of AI-assisted care) more complex. As such, if AI-CDSS is to be used in healthcare, attention needs to be paid to their potential to disrupt current understandings of professional responsibility and accountability. Professional regulation needs to be updated to accommodate this new actor in the clinical space, and professional guidelines are needed to steer clinicians towards the appropriate use of this new actor. The Royal College of Physicians^17^ and The Royal College of Radiologists^18^ have called for working with regulators, NICE, NHS England and NHS Digital to develop standards and guidance for AI. Additionally, The Society of Radiographers’ AI working group^19^ has published guidance for clinical imaging and therapeutic radiography professionals. This comprises, however, recommendations for a single clinical professional group, which fall short of what is needed: namely enforceable standards which encompass and standardise all UK-regulated clinicians across the board.

### Practice underpinned by professional guidance

It seems unlikely that an AI-CDDS would be deployed into clinical areas without additional workforce education, training and the creation of AI-specific clinical roles. Report 2 stipulates the need for new roles of ‘digital and AI specialist clinicians’, who ‘communicate effectively with technical specialists like data scientists, liaise with clinical teams, promote safety and ensure products deliver real clinical impact’ (NHS AI Lab & Health Education England, p. 67).^10^ These new roles will require training and would be intended to bridge gaps between ‘creators’ (of AI systems) and (clinical) ‘users’. They would insert specialist expertise into the clinical space, perhaps even creating a new clinical subspecialty. But the creation of these roles would merely shift the problem of the gap in guidance rather than solving it. The introduction of a new specialist clinical role will require regulators to accommodate that role and consider the impact on the profession (including what counts as appropriate reliance on new technology, and the new professional dynamic created by a role that interfaces between human and non-human actors). Professional guidance will still be needed for the new specialty, and non-specialist clinical users will still need guidance, at least, to set out under what conditions the specialty can and should be consulted.

This will all require careful oversight to ensure clinical safety for patients, professional safety for clinical practitioners and reputational safety for the institution of healthcare, where:

- *clinical safety* would require that all clinical professionals have training to understand how an AI-CDSS application may benefit patients and how it could harm them;
- *professional safety* would require that the clinician knows what is expected of their practice with regard to the AI-CDSS, and that they are given clear instructions about the parameters of AI-CDSS use and misuse;
- *reputational safety* would require the protection from reputational risk to clinical institutions from the damage caused by avoidable and predictable harms arising from AI-CDSS use.

To ensure clinical, professional and reputational safety, clinicians will need comprehensive training in the technologies presented to them to use, and authoritative and accessible guidance to help them navigate this newly complicated clinical space, enabling them to work safely with their newly presented digital tools. Report 2 says that users should ‘[u]se AI within healthcare settings in accordance with guidelines’ (NHS AI Lab & Health Education England, p. 105),^10^ and report 1 notes that “clinicians look to regulators for guidance on how they should use AI technologies and for reassurance that using AI in clinical practice will not threaten their professional registration” (NHS AI Lab & Health Education England, p. 34).^9^ Report 1 also recognises the lack of such guidance, arguing that for confidence to be developed in the use of AI in healthcare setting, professional guidelines are needed around ‘creating, implementing and using AI for all clinical staff groups’ (NHS AI Lab & Health Education England, p. 14).^9^ Confusingly, however, report 2 implies that usable guidelines exist, saying that users should follow ‘any developed guidance from regulators of healthcare workers on the development and use of technology including AI’ (NHS AI Lab & Health Education England, p. 40).^10^ Report 2 also notes that users need to have ‘[a]wareness of issues relating to personal and organisational liability for AI technologies’ (NHS AI Lab & Health Education England, p. 41)^10^—which is unhelpful given that, as report 1 acknowledges, ‘[c]urrently, there is uncertainty as to who will be held to account if AI products are used to make clinical decisions that lead to patient harm’ (NHS AI Lab & Health Education England, p. 9),^9^ and there is certainly no clarity in UK law.^20^

This is all to say that professional regulators need to consider how they should regulate the use of AI in the clinical space, and professional guidance around the use of AI-CDSS in clinical practice needs to be drafted and released. Until then, it seems that clinical users will have to do what is suggested by report 2 and use generic principals of good practice ‘for example, applying the principles of Good Medical Practice or Good Scientific Practice’ (NHS AI Lab & Health Education England, p. 41).^10^ This does feel like asking them to make it up as they go along, however, and the absence of prescribed practice standardisation risks both inconsistent and dangerous practice.

With this in mind, let us now examine why it is entirely unsuitable to use existing standards and non-specific principles of good practice when AI-CDSSs are used.

### The need for AI-CDSS-specific guidance

Specific guidance to address the use of AI-CDSS is needed because the technology reflects such a significant change in healthcare delivery. As described above, existing clinical professional regulation regulates both professional-patient relations and intraprofessional relations. Where it pertains to the use of technology, guidance assumes passive tools that operate in fixed and predictable ways. The kind of AI-CDSS that we are considering here, which has evolving functionality and which plays an active role in decision-making, is neither a passive tool nor a fellow professional—but occupies some as yet undefined liminal space in-between.

Beyond the scope of this paper, but important to its context, is the regulation of AI-CDSSs themselves. If AI-CDSSs are adopted as medical tools, then priority must be given to developing robust technology assessment frameworks before permitting any deployment in a clinical environment. Evidence of AI-CDSS safety and accuracy must be prerequisite for clinical use, underpinned by device regulation. The quality of an AI-CDSS needs to be clear to users, requiring labelling that encompasses system transparency, explainability, safety, etc. This must be in place before we can develop professional clinical guidelines. Poor quality AI-CDSSs will not be eliminated by professional guidance and their presence will serve only to increase the challenges faced by clinical users employing AI-CDSSs.

It might be argued that there are already high-level guidelines around professional conduct, or use of intelligent technologies, which could be transposed and used to inform practice regarding AI-CDDSs. This may be true, but the former will be so high-level it will be functionally useless, requiring such a significant level of interpretation that they cease to guide.

We know, from principles of Good Medical Practice,^11^ that clinical decision-making should be evidence based and communicated to patients in a way they can understand. But, given that AIs can be ‘black boxes’ in regard to their reasoning, it is unclear how this latter obligation be operationalised; as the clinician will not be able to fully understand, and thus articulate, the basis of any AI-CDSS-based recommendation. While ‘explainability’ is frequently offered as an important principle for good AI use,^21 22^ it is challenging to elicit an adequate level of explanation for the basis of outputs from those AI-CDSSs built on machine learning. To the extent that the reasoning behind AI-CDSS recommendations remains opaque, therefore, questions arise about appropriate clinical use, for example, should patients be given the choice between an explainable course of action and an unexplainable alternative that is potentially more optimal? Either way, perhaps a precautionary approach should be adopted, whereby only clinicians with proven knowledge of the clinical specialty in question be permitted to use AI recommendations?

Existing professional regulation requires clinical professionals to be respectful of colleagues, accept responsibility for mistakes, act to prevent unsafe colleagues from harming patients, operate duty of candour, and not bring the profession into disrepute. However, such stipulations assume that the clinician is practising with other human clinical agents. Where an AI-CDSS is functioning as an active advice giver, should it be treated as a tool or more like a colleague? Should the clinician own mistakes made by the AI-CDSS and take responsibility for them, or should some other actor—for example, the AI-CDSS’s creator—be (perhaps jointly) liable? How can the profession act to maintain its reputation and avoid disrepute if clinical decision-making too blindly follows the AI-CDSSs recommendation—or fails to follow it when it should?

Existing practices for smart clinical machinery, for example, for the use of ‘INTELLiVENT’^23^ (an intelligent ventilator mode that monitors intubated patients and titrates settings based on continuous feedback) could be borrowed, which would tell us that the system needs to be monitored by a clinician and requires near-constant human oversight. But what level of monitoring and oversight is required of AI-CDSS that is designed to be able to see patterns and make diagnoses, and therefore propose treatment pathways, that a human would or could not, and which cannot explain is decision-making process? Under what*—if any*—circumstances should an AI-CDSS recommendation be blindly trusted or overridden? We presume that clinicians will pay close attention to AI-CDDS outputs before they are acted on, but systems that work consistently well may become overly relied on. This is problematic, as a clinician’s atrophy of vigilance may result in an erroneous AI-CDDS output being used and a patient being detrimentally affected. How can this potential atrophy of vigilance^24^ be manged? These issues collectively indicate the need for specific guidance to inform both clinical practice and societal expectations regarding AI-CDSS use in clinical care.

It is clear that no existing guidelines or regulatory frameworks have the specificity required to effectively steer the use of AI-CDSSs in clinical decision-making. And without clear professional, ethical and legally justifiable guidance, individuals or the organisations for which they work, will be left to form their own interpretation of how to apply generic standards of clinical practice. This will, at best, lead to variations in approaches to, and quality of, care which patients receive when AI-CDSSs are employed at the bedside, which will undermine justice in healthcare, as like would not be treated as like.^20^ Hence, specific guidance is needed, and, to borrow from Huxtable (commenting on COVID-19 guidance) ‘authoritative national ethical guidance should help to bring clarity, consistency and fairness to decision-making’.^25^

### The case for starting now

Insofar as the argument above is compelling, and we accept that no existing guidelines or regulations can do the job, then there is a significant gap which will create an unreasonable burden on clinicians. It is unfair to ask clinicians to prepare to use novel technologies without professional guidance, as without it they are ill equipped to anticipate, and thus avoid, practical, ethical and legal issues. Clinicians should not be expected to work it out as they go along; they deserve to know that their clinical practice is lawful and conforms to agreed standards of professional practice.^26^

Clinical users must know the regulatory and liability implications of AI-CDSSs in advance of them being deployed. For this reason, we cannot permit a situation where professional guidance is announced after an AI-CDSS has been operationalised. Such a late arrival would disrupt the orderly adoption of AI-CDSS by potentially requiring fundamental changes in practice just at the point of deployment. Rather, development and deployment of AI-CDSSs ought to be informed by the professional guidance that will be followed, rather than having to retrofit its adoption.

Creating guidance will be challenging, however, as (as noted by Topol Review) the healthcare workforce will need to be prepared “for jobs that have not yet been created, technologies that have not yet been invented and problems that we don’t yet know will arise” (The Topol Review, p. 21).^27^ Thus, targeted professional guidance for users of individual AI-CDSS applications will need to be iterative and have scope to develop once AI-CDSSs have been deployed into healthcare and the issues and challenges move from the hypothetical to the practical. This will require an organised and unified approach.

### A unified approach to guidance?

To avoid inconsistency between the various clinical professions, professional guidance for the use of AI-CDSSs should be drafted in the form of a single document composed of a united voice between the regulating and professional bodies, and even involving key clinical trade unions, for example, the Royal College of Nursing, British Medical Association and UNISON.

Unified guidance is necessary to avoid the conflict and confusion that might arise from independent standards with different goals. A united and uniform approach resulting in the collaborative release of harmonised guidance will permit better integration of that guidance into universal healthcare practices. This, in turn, will benefit patients who will be able to more easily predict what to expect from any given healthcare professional using AI-CDSSs, rather than having separate practices from each profession, all underpinned by different guidance documents.

While professional codes of conduct have traditionally been created and kept in-house, there is precedent for the collaborative framing of guidance around single issues: a recent example being clinical regulation during the early part of the COVID-19 pandemic.^28^

As clinical working has developed over time, there has been a shift towards non-exclusivity in skills acquisition and practice where members of different professions acquire roles that were once the dedicated remit of single professions: for example, drug prescribing being now undertaken by appropriately qualified non-medical clinical professionals. If AI-CDSSs are to aid all clinicians, then the professional guidance supporting its use needs to be flexible enough to adapt to any role and any level of practice seniority; thus, they need to be developed collaboratively by and for all those who are affected.

To support the future use of AI in practice, HEE has developed a Healthcare Technologies Capability framework^29^ but, this does little to address the missing professional regulatory guidance specific to AI-CDSS use. Indeed, it vaguely states that ‘legislation may not keep pace with technological innovation’ and that ‘where clear regulations do not exist’ users are to ‘aim to apply the ethical principles of beneficence, non-maleficence, autonomy and justice as a guide to using digital technology (eg, promoting principles of privacy, confidentiality and equality)’.^30^ While this framework could be used to construct clinical training curricula with useful and well-considered content, it cannot cover the professional principles required by the clinical regulators because those principles do not yet exist. As such, any proposed curriculum runs the risk of inadvertently advising practice that would put professionals at risk if ever their practice came to be questioned by their clinical regulator. This problem has the scope to be multiplied if each education provider interprets the HEE framework differently. Unified professional guidelines would help by providing professional structure, certainty and substance to the educational programmes that report 2^10^ outlines. Additionally, a congenial approach to interprofessional working regarding the use of AI-CDSSs in healthcare would be more readily achievable if all courses were underwritten by the same professional guidelines.

### What could professional guidance contain?

Any guidance would need to be loose enough to envelope any AI-CDSS that may come into the healthcare environment, but restrictive enough to be effective in defining professional clinical misconduct so that it can be recognised and addressed.

Elements that could be considered may include:

- Principles, expectations and obligations of collaborative working with AI-CDSS creators (as well as one’s conventional multidisciplinary team).
- The requirement for the possession of the knowledge and skills (and specification of such knowledge and skills) to safely use the AI-CDSS in question.
- Rules to ensure that the AI-CDSS is safe to use, and that related issues (eg, data bias, drift, brittleness) are accounted for and mitigated against.
- Guidance and standards around the reporting of issues with any AI-CDSS, as and when they arise.
- Guidance regarding the thresholds of when clinicians can or should decline to use the AI-CDSS and revert to their own knowledge base to provide patient care.

Aside from serving to guide clinical users, the development of unified guidance could be used as an opportunity to define key fundamental principles of AI-CDSS adoption to clinical use.

One such principle could be to end the uncertainty of who holds responsibility for the use of AI-CDSS in clinical practice. As per the example above, for instance, if an AI-CDSS causes patient harm, there is scope for guidance to recommend that responsibility is shared between the AI-CDSS’s creator and clinical users, instead of either the clinician or the AI-CDSS creator being held singularly responsible for that outcome. Clinicians could also undertake to report problems as they arise, thus promoting a contemporaneous response from its creator, allowing for a collaborative approach to problem solving in real-time. If problems are undetected by both users and creators, both groups could undertake to share responsibility for negative outcomes and subscribe to a joint model of restitution to amend for harms caused using, for example, a shared indemnity arrangement.^20^ (There are complex questions here about how this would be possible in a fast-paced clinical environment, how it would be reimbursed and to what extent it may be used to roll out systems prematurely, working through issues after AI-CDSS deployment. This returns us to our earlier point of the necessity of adequate regulation prior to the release of AI-CDSS, and raises the challenging question: how good ought an AI-CDSS be before its use can be permitted in clinical practice? What constitutes a minimally viable product, which would satisfy the demands of clinical users, regulators and patients, remains a critical question.)

## Conclusion

This paper has highlighted the lack of professional guidance for clinicians who will use AI-CDSSs in performance of their healthcare roles and argued that this is a problem for ensuring clinical safety for patients, professional safety for clinical practitioners and reputational safety for healthcare. A united and standardised approach to guidance development between the regulating bodies and potentially the key clinical trade unions is preferable to individualistic approaches. This would benefit patient care by promoting the harmonious adoption of AI-CDSS with a streamlined approach for all healthcare practitioners.

## Footnotes

Individuals can hold more than one archetype position. For example, a user could also be involved in developing an artificial intelligence (AI) application, thereby also being a creator. The complex array and interplay of roles that would be needed for an AI application to reach clinical care is recognised here but will not be further explored as this paper is focused on the professional needs of clinicians.
